# A Natural Antibacterial-Antioxidant Film from Soy Protein Isolate Incorporated with Cortex *Phellodendron* Extract

**DOI:** 10.3390/polym10010071

**Published:** 2018-01-13

**Authors:** Shumin Liang, Lijuan Wang

**Affiliations:** 1 Key Laboratory of Bio-Based Materials Science and Technology of Ministry of Education, Northeast Forestry University, Harbin 150040, China; liangshumin@nefu.edu.cn; 2 Research Center of Wood Bionic Intelligent Science, Northeast Forestry University, Harbin 150040, China

**Keywords:** food packaging, soy protein isolate, cortex *Phellodendron* extract, antibacterial activity, antioxidant activity

## Abstract

An active film was prepared by incorporating cortex *Phellodendron* extract (CPE, an active agent) into a soybean protein isolate (SPI). Different concentrations of CPE (0%, 10%, 12.5%, 15%, 17.5%, 20%, or 22.5%, *w*/*w*, based on SPI) were mixed into the films characterized by Fourier transform infrared spectroscopy, X-ray diffraction, scanning electron microscopy, thermogravimetry, tensile tests, and barrier properties. The rheological properties of the solutions were also tested. The effects of the CPE content on the antibacterial and antioxidant activities of the films were examined. The results indicated that new hydrogen bonds formed between molecules in the films, and the crystallinity of the films decreased. The incorporation of CPE had no significant influence on the thermal stability of the films. Films containing 15% CPE had the maximum tensile strength of 6.00 MPa. The barrier properties against water vapor, oxygen, and light enhanced with the incorporation of CPE. The antioxidant activity of the SPI film was also improved. The films were effective against *Staphylococcus aureus* (*S. aureus*, Gram-positive bacteria). These results suggest that the SPI/CPE film can potentially extend the shelf lives of foods.

## 1. Introduction

Plastic materials which are easily processed and versatile are widely used for food packaging [1]. However, plastics impose problems because they do not easily degrade in the environment [2]. Therefore, biodegradable films will potentially reduce or eliminate some traditional polymeric packaging materials. Recently, biodegradable materials such as proteins, polysaccharides, and lipids have attracted considerable interest for use in food packaging [3].

Soybean protein isolate (SPI) is an abundant and inexpensive raw material with nutritional properties [4]. SPI films have moderate mechanical properties and good barrier properties against oxygen, and are impermeable to lipids [5]; thus, they can be used for food packaging [3]. However, SPI is very easily contaminated by bacteria. Therefore, it is important to endow SPI-based films with antibacterial properties to prolong the shelf lives of foods [6].

Food preservation with natural additives has become very popular because these additives are “generally recognized as safe” [7]. The incorporation of active agents into films can maintain their activities for long periods of time. Many natural antibacterial compounds are derived from plants, including basil, cinnamon, thyme, oregano, clove, and rosemary [7]. Berberine is an isoquinoline-type alkaloid derived from plants, with antidiabetic, antidiarrheal, antimicrobial, immunostimulatory, blood-pressure-lowering, and anti-inflammatory properties. The strong bacteriostatic activities of berberine against bacteria have been investigated in many studies [8]. Cortex *Phellodendron* (CP) derived from *Phellodendron amurense* Rupr., an inexpensive and abundant herb. There are a number of alkaloids (berberine, palmitine, and phellodendrine) [9] in CP, and they are known as anti-inflammatory agents [9]. Some studies have reported that the anti-inflammatory properties [9] and neuroprotective effects [10] of CP. The high-value components of cortex *Phellodendron* (e.g., berberine) can be extracted with the hot-water method [11]. In in vitro tests, cortex *Phellodendron* extract (CPE) showed antioxidant activity and inhibitory effects on bacteria.

The objective of this study was to prepare a natural antibacterial-antioxidant film. The effects of CPE concentration on film activities (antibacterial and antioxidant activities) were determined. Physical properties (mechanical, water vapor permeability (WVP), oxygen permeability (OP), and optical) were examined. The composite films were also analyzed with Fourier transform infrared (FTIR) spectroscopy, X-ray diffraction (XRD), scanning electron microscopy (SEM), and thermogravimetric analysis (TGA). The rheological properties of film-forming solutions (FFSs) were also measured.

## 2. Materials and Methods 

### 2.1. Materials

SPI (soy protein isolate, more than 90% protein content on dry basis) was obtained from Harbin High Technology Food Co., Ltd. (Harbin, China). Glycerol, sodium hydroxide (NaOH), sodium carbonate (Na_2_CO_3_), and ethanol were obtained from Liaoning Quan Rui Reagent Co., Ltd. (Jinzhou, China). Foline-Ciocalteu regent and 2,2-diphenylpicrylhydrazyl (DPPH) were provided by Shanghai Xirun Reagent Co., Ltd. (Shanghai, China).

### 2.2. Preparation of CPE

The preparation of CPE was slightly modified according to a previous study [10]. Firstly, the cortex *Phellodendron* was crushed into powder with a size of 60–100 mesh. Then, the powder (100 g for 1000 mL deionized water) was extracted for 2 h at 100 °C after soaking 24 h. Finally, the mixed solution was centrifuged at 2000 rpm for 5 min to remove the impurities. The yield of the CPE was 1.30 wt %.

### 2.3. Preparation of SPI and SPI/CPE Films

Firstly, 6 g of SPI and 3 g of glycerol were dispersed in 100 mL of solution containing CPE. The CPE was added at 0%, 10%, 12.5%, 15%, 17.5%, 20%, or 22.5% (*w*/*w*, based on SPI) denoted as SPI-E0, SPI-E10, SPI-E12.5, SPI-E15, SPI-E17.5, SPI-E20, and SPI-E22.5, respectively. The mixed solution was stirred for half an hour, and the pH value was adjusted to 9 by using NaOH solution (2 mol·L^−1^). After that, the solution was heated for 20 min at 85 °C. Finally, the FFS was cast into a Plexiglas plate (290 mm × 280 mm × 50 mm) and then dried at 55 °C for 12 h in a vacuum drying oven. The dried film was maintained at a humidity of 43% and 25 °C for 24 h before further tests.

### 2.4. Characterization

#### 2.4.1. FTIR Spectroscopy

FTIR spectra of the CPE-free and blended films were determined by a Thermo Nicolette 6700 spectrophotometer (Thermo Fisher Scientific Co., Ltd., Waltham, MA, USA). All the spectra of films were collected from 4000 to 500 cm^−1^ at a resolution of 4 cm^−1^.

#### 2.4.2. XRD Analysis

The XRD patterns of the films were recorded by a X-ray diffractometer (D/max-2200) with Cu Kα radiation at 40 kV and 30 mA. The scanning scope of 2θ was in the range from 5° to 50° at a scan speed of 5°·min^−1^.

#### 2.4.3. SEM Observation

Firstly, the samples were coated with a thin gold layer. Then, the micrographs of surface and cross-section of the films were obtained by using SEM (Philips-FEI Co., Amsterdam, The Netherlands) at an acceleration voltage of 5 kV.

#### 2.4.4. TGA Analysis

The samples were analyzed by a TA Instruments TGA Q500 (New Castle, DE, USA) in the range from 25 to 600 °C at a rate of 10 °C·min^−1^. The curves of thermogravimetric analysis (TGA) were obtained, and the first-order derivative of TGA curves was expressed as differential thermogravimetric analysis (DTG).

#### 2.4.5. Rheological Measurement

Rheological properties of the FFS (SPI, CPE, and SPI/CPE) were examined by using a rheometer (AR2000ex, TA Instruments, Newcastle, DE, USA) equipped with a cone and plate geometry with a cone angle of 2°, a diameter of 40 mm, and a gap of 57 μm. Viscosity was tested at a shear rate of 0.1–100 s^−1^. Flow behavior was described by the Ostwald-de Waele model (Equation (1)):

(1) $$\tau =\kappa {\dot{\gamma }}^{n}$$

where *τ* is the shear stress (Pa), *κ* is the consistency coefficient (Pa·s*^n^*), $\dot{\gamma }$ is the shear rate (s^−1^), and *n* is the flow behavior index.

Dynamic oscillatory data were obtained at strain of 0.5% and the angular frequency from 0.1 to 100 rad·s^−1^. The storage modulus (*G*’, elastic behavior) and loss modulus (*G*’’, viscous behavior) were collected to describe the viscoelasticity.

#### 2.4.6. Mechanical Testing

The tensile strength (TS) and elongation to break (E %) of SPI-based films were measured with a tensile tester (XLW-PC, PARAM, Jinan, China). The tensile rate was set to 300 mm·min^−1^. The samples (80 mm × 15 mm) were prepared for the test.

#### 2.4.7. WVP Measurement

The film (16.6 cm^2^ round shape) was pasted on the mouth of the test cup containing 20 g anhydrous calcium chloride (CaCl_2_) at 0% relative humidity (RH), then the cups were placed into a desiccator containing NaCl saturated solution at room temperature and 75% RH. The weight change of the sample was measured until it reached equilibrium. WVP of the films was calculated according to Equation (2):

WVP = (∆*m* × *d*)/(*A* × ∆*t* × ∆*P*) (2)

where ∆*m* is the weight difference (g), ∆*t* is the time difference (s), *d* is the film thickness (m), *A* is the exposed film area (m^2^), and ∆*P* is the water vapor partial pressure (Pa).

#### 2.4.8. OP Measurement

OP was analyzed through the Chinese National Standard (GB/T 19789-2005). An Ox-Tran equipment (PERME OX2/230, Labthink, Jinan, China) was used to determine the OP values at 25 °C and 0% RH.

#### 2.4.9. Color and Light Transmission Analysis

The color of SPI-based films was indicated by L* (lightness/brightness), a* (redness/greenness) and b* (yellowness/blueness) through a portable colorimeter (Xrite2600d; X-Rite Inc., Grand Rapids, MI, USA). Light transmittance of the samples was measured at wavelengths between 200 and 600 nm by using an ultraviolet-visible (UV–Vis) spectrophotometer (UV-2600, Shimadzu, Kyoto, Japan).

#### 2.4.10. Determination of Antibacterial Activity

*E. coli* (ATCC 25922-3) and *S. aureus* (ATCC 25923-3) were used to evaluate the antibacterial activities of CPE and SPI-based films with disc diffusion method. CPE (200 µL) was poured into the hole (diameter of 10 mm) on the agar plates. The films were cut into disk shape with a diameter of 10 mm and placed on new medium agar plates containing about 10^3^–10^4^ CFU·mL^−1^ of tested bacteria. All plates were then incubated for 24 h at 37 °C. The antibacterial effects of the films were expressed by the diameter of the inhibition zone. All samples were tested in triplicate.

#### 2.4.11. Evaluation of Antioxidant Activity

The release of antioxidant agents from the films was performed by the following method. The samples of 0.05 g were immersed into a 10% (*v*/*v*) ethanol solution (10 mL) in conical flasks. Then, the flasks were shaken at 100 rpm and 25 °C.

The total phenolic content (TPC) of the film was examined by the Foline-Ciocalteu method with minor modifications [12]. Firstly, film extract (1 mL) was mixed with Foline-Ciocalteu regent (4 mL) for 3 min in the dark at room temperature. Then, 7.5% (*w*/*v*) of sodium carbonate solution (5 mL) was added for 2 h. Finally, the absorbance of the resulting solution was monitored at 765 nm with a spectrophotometer (UV-2600, Shimadzu, Kyoto, Japan). The TPC in the film was expressed as gallic acid equivalents. The TPC of the samples was calculated as follows:

TPC (mg g^−1^) = (*c* × *V*)/*m* (3)

where *c* is the quantification of gallic acid on the basis of a standard curve (mg·L^−1^), *V* is the volume of film extracts (L), *m* is the weight of dried film (g).

The resulting solution was prepared by mixing film eatract (1 mL) and DPPH solution (4 mL, 25 mg·L^−1^; Ruitaibio Company, Beijing, China) for half an hour in the dark at room temperature. Then, the absorbance of the sample was measured at 516 nm. The activity was expressed as follows:

DPPH scavenging effect (%) = [(Abs_DPPH_ − Abs_film extract_)/Abs_DPPH_)] × 100 (4)

#### 2.4.12. Statistical Analysis

Statistical data analyses were performed by using SPSS software (SPSS Inc., Chicago, IL, USA). Duncan’s multiple range test was used to evaluate significantly (*p* < 0.05) different means.

## 3. Results and Discussion

### 3.1. FTIR Spectroscopy of Films

**Figure 1:**
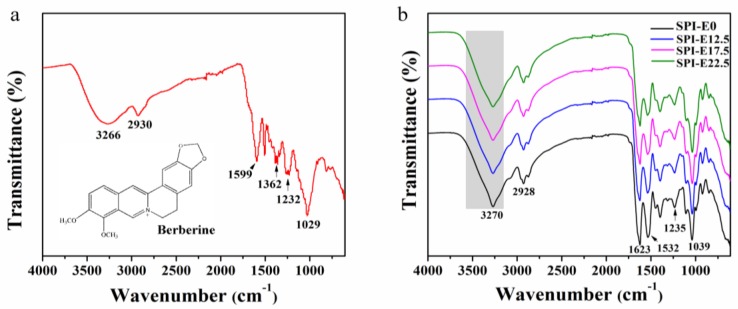
Fourier transform infrared spectroscopy (FTIR) spectra of (**a**) cortex *Phellodendron* extract (CPE) and (**b**) soybean protein isolate (SPI)-based films.

The FTIR spectrum of CPE is exhibited in Figure 1a. The characteristic absorption band at 3266 cm^−1^ corresponds to phenolic groups. The bands at 1599 and 1029 cm^−1^ are assigned to the stretching vibrations of the C=C aromatic ring and the methoxy group [13]. The spectrum confirms that berberine is present in the CPE [14].

As shown in Figure 1b, the spectra of the SPI films show an absorption band at 3270 cm^−1^, corresponding mainly to the free O–H groups and amine N–H stretching of the SPI in the films [15]. The other absorption bands correspond to C=O stretching (amide I) at 1630 cm^−1^, N–H bending (amide II) at 1532 cm^−1^, and C–N stretching (amide III) at 1235 cm^−1^. The peak at around 2928 cm^−1^ arises from the asymmetric stretch vibrations of =C–H and –NH_3_^+^ [16]. The characteristic peak at around 1039 cm^−1^ is attributed to C–H and C–O–H deformations. In the range of 800–1150 cm^−1^, the major absorption bands are related to glycerol [17]. With the CPE increased, the amide I slightly shifted from 1623 to 1625 cm^−1^, indicating that there were interactions between polymer molecules in the films [18]. The peak at 3700–3100 cm^−1^ became slightly broader, which further proved that hydrogen bonding interactions formed between the molecules in the film [19].

### 3.2. XRD Patterns of Films

**Figure 2:**
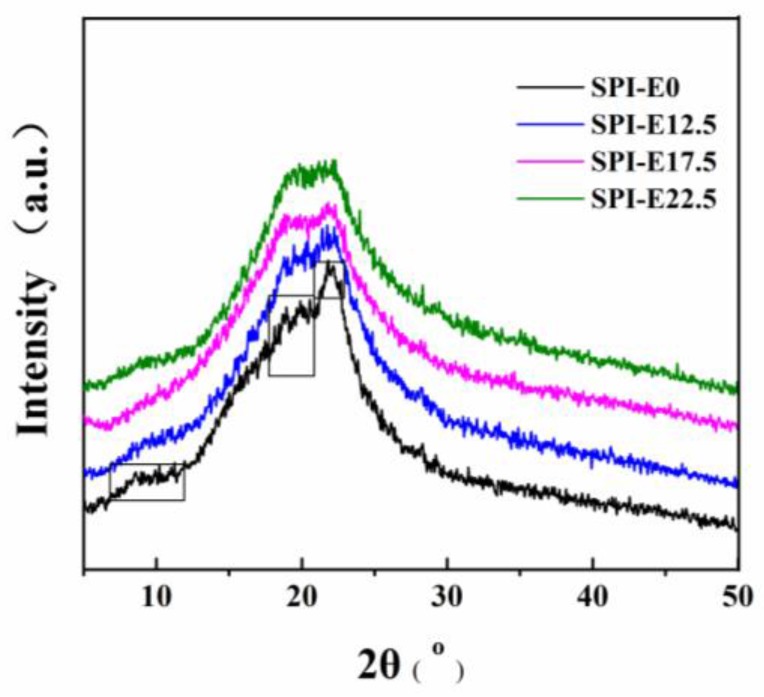
X-ray diffraction (XRD) patterns of the SPI-based films.

The XRD patterns of the CPE-free film and SPI/CPE films are shown in Figure 2. The peaks for the CPE-free film appeared at 2θ values of 8.9° and 19.9°, reflecting the α-helix and β-sheet structures of the SPI secondary conformation, respectively [20]. The CPE-free film showed a dominant amorphous halo and a broad peak at 2θ of around 21.8°, indicating that amorphous globulin (7S and 11S) was the important part in film [21]. With the incorporation of CPE, new peaks did not appear, suggesting a good compatibility between SPI and CPE. The peak intensity decreased as the CPE content increased, indicating that the crystalline structure of SPI was disrupted to some extent [22].

### 3.3. Morphologies of the Films

**Figure 3:**
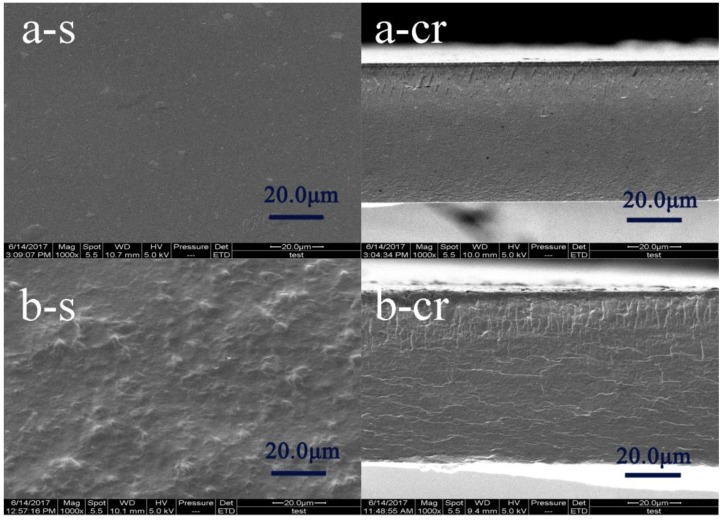
Scanning electron microscopy (SEM) micrographs of the surface (s, **left**) and cross-section (cr, **right**) of the (**a**) SPI and (**b**) SPI/CPE films.

The morphologies of the surface and cross-section of the samples are exhibited in Figure 3. The CPE-free films were smooth and compact. However, the addition of CPE made the films rougher and less compact. There were some large particle-like structures on the surface and some folds on the cross-section of the films, suggesting that CPE may disrupt the protein network [23]. These structures may have reduced the strength of the film and may explain—at least in part—why CPE caused a decrease in the TS at concentrations higher than 15%.

### 3.4. Thermal Analysis

**Figure 4:**
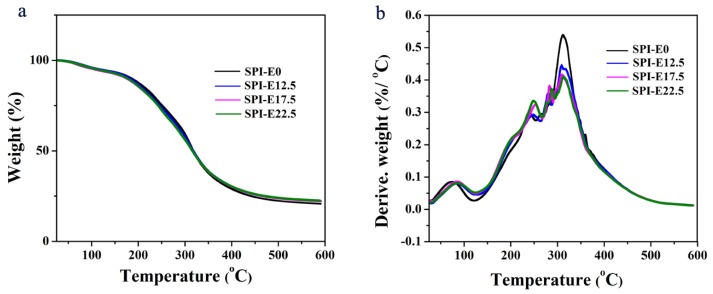
(**a**) Thermogravimetric analysis (TGA) and (**b**) differential thermogravimetric analysis (DTG) curves of the SPI-based films.

The TGA (a) and DTG (b) of the samples are shown in Figure 4. In the films, the weight loss as the temperature increased from 30 °C to 120 °C was mainly related to the loss of water. The weight loss at 120–260 °C was probably attributed to the evaporation of glycerol, and the weight loss at 300–400 °C could be the degradation of the proteins [24].

With the incorporation of CPE, there were no obvious changes in the TGA curves. In the DTG curves, the maximum decomposition temperature in the first stage increased from 75.13 to 87.51 °C as the CPE content increased in the SPI-based films. This could have resulted from the interaction between the molecules [25]. Weight loss from 260 to 300 °C was attributed to the thermal degradation of CPE, whereas no weight loss occurred in the CPE-free film. The maximum decomposition temperature for all the SPI-based films did not change markedly. Therefore, the thermal stability of the films had little change with the incorporation of CPE.

### 3.5. Rheological Properties of FFS

**Figure 5:**
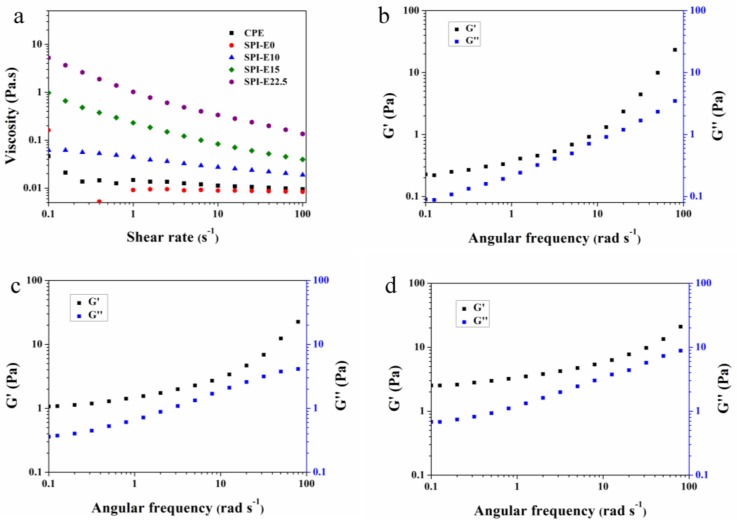
(**a**) Changes in viscosity of blended solution with different CPE content. Dynamic rheological properties obtained at 0.5% strain for blended solution: (**b**) SPI-E12.5; (**c**) SPI-E17.5; and (**d**) SPI-E22.5.

Figure 5a shows the effect of CPE concentration on the viscosity of the FFS. At a shear rate of 4 s^−1^, the viscosity of the SPI-E0, SPI-E10, SPI-E15, and SPI-E22.5 FFS were 0.009, 0.03, 0.12, and 0.49, respectively. According to molecular chain theory, there was more entanglements between SPI and CPE molecules at higher concentration of CPE. As a result, the movement of molecules in mixed solution was more difficult [26]. On the other hand, the average molecular weight of the component was higher with the addition of CPE. For macromolecules, the hydrodynamic volume increases with higher molecular weight, resulting in a greater thickening power [27]. The viscosity of SPI-E10, SPI-E15, and SPI-E22.5 solutions decreased gradually with increased shear rate (between 0.1 and 100 s^−1^), which was a shear-thinning behavior (non-Newtonian behavior). Conversely, the CPE-free solution exhibited a Newtonian behavior. The solution containing CPE had a higher viscosity than CPE-free solution at a higher shear rate. This result may be related to the formation of new hydrogen bonds between SPI and CPE, which was beneficial for enhancing the tensile strength of the films [28].

As shown in Table 1, the Ostwald-de Waele model could be used to describe the rheological properties of the FFS due to the value of *R*^2^ more than 0.99. According to the Ostwald-de Waele model, the solution could be divided into pseudoplastic fluid with shear-thinning nature (*n* < 1), Newtonian fluid (*n* = 1), and dilatant fluid with shear thickening nature (*n* > 1). The n values of SPI-E10, SPI-E15, and SPI-E22.5 solutions were 0.835, 0.662, and 0.584, respectively. Therefore, all of the mixed solutions showed a shear thinning behavior, indicating that the mixed solution was a pseudoplastic fluid (non-Newtonian fluid). However, the CPE-free solution exhibited the highest value of n, which tended towards 1 (Newtonian fluid). The consistency index (κ, Pa·s*^n^*) increased from 0.010 to 0.920 with the incorporation of CPE from 0% to 22.5%, showing that the κ was proportional to the concentration of CPE in the SPI-based solution. So, the influence of CPE was dominant in the viscosity of FFS.

The effects of CPE content on elastic behavior (*G*’) and viscous behavior (*G*”) are shown in Figure 5b–d. For all samples, the *G*’ and *G*” increased with increasing angular frequency. Additionally, *G*’ was higher than *G*” at angular frequency from 0.1 to 100 rad·s^−1^, and elastic behavior was higher than sticky behavior. This is a typical phenomenon, where the rearrangements of a network of droplets does not have enough time to adapt to strain during a single oscillation [29]. This result suggested that mixed solutions were weakly elastic gels, which provided a solid-like structure, and that this structure could restrict the movement of droplets [30].

### 3.6. Mechanical Properties of the Films

As shown in Table 2, TS and E % of the CPE-free film were 5.57 MPa and 130.13%, respectively. With the incorporation of CPE at 15%, the TS of the composite film increased from 5.57 to 6.00 MPa. However, TS gradually decreased when the addition of CPE exceeded 15%. When the CPE concentration reached 22.5%, TS decreased to 4.50 ± 0.16 MPa (*p* < 0.05). These results are attributed to the intermolecular interactions among CPE, SPI, and glycerol. A decreasing trend in E% was observed, indicating that the CPE addition could reduce the ductility and flexibility of the films.

### 3.7. WVP

The WVP values for the samples are shown in Table 2. The CPE-free film had the highest WVP value of 2.56 × 10^−10^ g·mm^−2^·s^−1^·Pa^−1^ because of its hydrophilicity [31,32]. As the CPE concentration increased from 0% to 22.5%, the WVP of the SPI-based films decreased from 2.56 to 2.44, 2.36, 2.34, 2.29, 2.24, and 2.19 × 10^−10^ g·mm^−2^·s^−1^·Pa^−1^. The higher CPE content may have led to smaller pores in the films, making the paths of the water molecules more tortuous [33]. The new hydrogen bonds between the SPI and CPE molecules would have also reduced the free hydrogen groups in the composite films [34]. These results may also be attributed to a reduction of the interstitial spaces in the protein matrix by water saturation, reducing the diffusion rate of the water molecules through the films [22]. A reduction in WVP is beneficial for extending the shelf lives of foods packed in the films.

### 3.8. OP

As shown in Table 2, the CPE-free film had the highest OP value of 0.18 × 10^11^ cm^3^·mm·m^−2^·day^−1^·atm^−1^. As the CPE content increased from 10% to 22.5%, the OP value decreased from 0.18 × 10^11^ to 0.08 × 10^11^ cm^3^·mm·m^−2^·day^−1^·atm^−1^—A 55.56% reduction. The SPI-based films were excellent barriers to nonpolar oxygen because of their hydrophilicity [23,35]. Some polar substances in CPE were mixed into the blended films, and therefore the condensing capacity of the nonpolar oxygen molecules in the films decreased [36]. The addition of CPE to the films may have caused a tortuous diffusion path such that the diffusion of oxygen through the blended films was more difficult.

### 3.9. Color and Light Transmission

As shown in Table 3, the color of the films was determined with the values for L*, a*, and b*. The composite films had lower value of L* and higher value of a* and b* than the CPE-free film. The L* values (lightness) decreased from 96.38 to 72.84, showing that the color of the films became darker. On the contrary, the b* values clearly increased from 10.67 to 93.94, indicating that the composite films changed to yellow. The a* values gradually became positive, suggesting a shift to redness.

**Figure 6:**
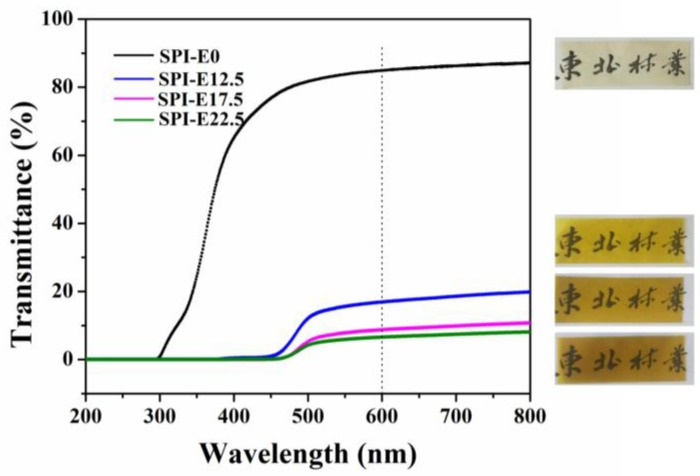
Light transmission of the SPI-based films.

The light transmission curves for the samples are shown in Figure 6. In the region of 200–380 nm, the light transmittance (T %) of the SPI/CPE films was 0%, but the CPE-free film had an extremely high value of T %, indicating that the incorporation of CPE in the films enhanced their UV barrier properties. The T % values of the CPE-free film and the SPI-E12.5, SPI-E17.5, and SPI-E22.5 films at 600 nm were 84.91%, 16.82%, 8.76%, and 6.57%, respectively. The transparency of the films was weak at higher levels of CPE. However, the blended films still retained good transparency and the words under the blended films could be clearly seen (Figure 6).

### 3.10. Antibacterial Activity

**Figure 7:**
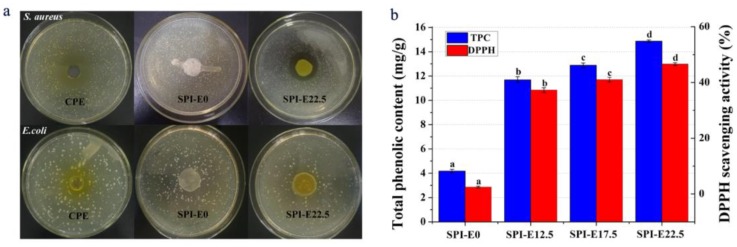
(**a**) The antibacterial activity of the CPE and films; (**b**) antioxidant activity including total phenol content (TPC) and 2,2-diphenylpicrylhydrazyl (DPPH) scavenging activity of the films.

The presence of *E. coli* or *S. aureus* in foods is a serious threat to safety. Therefore, these bacteria are used as indicators of food safety. The *E. coli* and *S. aureus* inhibition zones on SPI-based films with different contents of CPE are shown in Figure 7a. The antibacterial activity was estimated from the size of the inhibition zone. There was no inhibition zone against *E. coli* on the CPE and the films. On the contrary, the discs of CPE and films containing CPE showed clear inhibition zones gainst *S. aureus*, and the inhibition zone increased as the content of CPE increased. The antibacterial activities of the SPI/CPE films resulted from the release of alkaloid compounds with significant inhibitory effects. The bactericidal mechanisms in the SPI/CPE films includes the breakage of the bacterial cell-surface structure, leading to Ca^2+^ and K^+^ release from cells, which inhibits or influences the activities of enzymes and inhibits DNA duplication, RNA transcription, and protein biosynthesis [37]. The inhibition zones for *S. aureus* were larger than those for *E. coli*. The antibacterial activity of the SPI/CPE films against *E. coli* was not obvious. In general, the presence of a lipopolysaccharide layer is related to the viability of Gram-negative bacteria, and may reduce the susceptibility of these bacteria to natural extracts [38].

### 3.11. Antioxidant Activity

The TPC of the samples is shown in Figure 7b. The results showed that the CPE-free SPI film had the lowest TPC of 4.17 mg·(gallic acid)·g^−1^ (dried film). The incorporation of CPE into the film significantly increased TPC. The SPI-E22.5 film had the highest TCP of 14.87 mg·(gallic acid)·g^−1^ (dried film) and was about 3.6 times greater than that of the CPE-free film. This result may also be associated with the mechanical properties of the blended films. The hydrogen bonds were formed between phenolic compound and soy protein isolate via hydroxyl groups and the –NH_3_^+^ groups of amino acids [39].

As shown in Figure 7b, the CPE-free SPI film had slight scavenging activity on DPPH (~2.51%). This result was related to the small peptides from the hydrolysate of soybean protein [40]. The DPPH scavenging effect enhanced significantly at higher levels of CPE concentration. As the CPE concentration increased from 0% to 22.5%, the DPPH radical-scavenging activity increased from 2.51 to 46.59%. The results indicated that the addition of CPE into SPI film could improve the antioxidant activity of the films.

## 4. Conclusions

A natural antibacterial-antioxidant film was prepared from SPI mixed with CPE. With the addition of CPE, the TS of the films increased slightly because of the molecular interactions between the molecules. The elongation to break of the films tended to decrease as the CPE content increased. The WVP and OP values decreased with increasing CPE. The light transmittance and L* values also decreased with increasing CPE, indicating that the CPE addition enhanced the UV barrier properties of SPI/CPE films. Viscosities of the FFS increased with the increase of CPE content, suggesting that there were new hydrogen bonds between SPI and CPE, which enhanced the tensile strength of the films. In particular, the blended films possessed considerable antibacterial effect against *S. aureus*. The antibacterial properties were greater in the films with higher levels of CPE. Moreover, the antioxidant activity was also significantly improved. This study indicates that the SPI/CPE film could potentially be utilized in food packaging and preservation.

## Figures and Tables

**Table 1:** Rheological parameters of the Ostwald-de Waele model for the film-forming solutions.

Sample Code	Ostwald-de Waele Model		
	*κ*	*n*	*R*^2^
CPE	0.014	0.923	0.999
SPI-E0	0.010	0.970	0.998
SPI-E10	0.040	0.835	0.999
SPI-E15	0.185	0.662	0.998
SPI-E22.5	0.920	0.584	0.997

**Table 2:** Tensile strength (TS), elongation to break (*E*), water vapor permeability (WVP), and oxygen permeability (OP) of the SPI-based films.

Sample Code	TS (MPa)	E (%)	WVP (g mm^−2^·s^−1^·Pa^−1^ × 10^−10^)	OP (cm^3^mm·m^−2^·day^−1^·atm^−1^ × 10^11^)
SPI-E0	5.57 ± 0.04 ^a^	130.13 ± 3.18 ^g^	2.56 ± 0.14 ^c^	0.18 ± 0.01 ^c^
SPI-E10	5.68 ± 0.28 ^a^	88.73 ± 4.97 ^f^	2.44 ± 0.12 ^b,c^	0.16 ± 0.04 ^c^
SPI-E12.5	5.70 ± 0.17 ^a^	73.07 ± 2.65 ^e^	2.36 ± 0.07 ^a,b^	0.13 ± 0.02 ^b,c^
SPI-E15	6.00 ± 0.40 ^a^	56.73 ± 2.22 ^d^	2.34 ± 0.13 ^a^	0.12 ± 0.02 ^b,c^
SPI-E17.5	5.01 ± 0.37 ^b^	49.00 ± 2.85 ^c^	2.29 ± 0.03 ^a,b^	0.10 ± 0.02 ^a,b^
SPI-E20	4.73 ± 0.02 ^b^	37.43 ± 0.05 ^b^	2.24 ± 0.04 ^a^	0.08 ± 0.01 ^a^
SPI-E22.5	4.50 ± 0.16 ^b^	31.00 ± 2.61 ^a^	2.19 ± 0.01 ^a^	0.08 ± 0.01 ^a^

Data were reported as mean ± standard deviation. Different letters indicated significantly different means (*p* < 0.05).

**Table 3:** Color properties of the films.

Sample Code	L*	A*	B*
SPI-E0	96.38 ± 0.11 ^a^	−1.80 ± 0.06 ^b^	10.67 ± 0.57 ^a^
SPI-E12.5	83.42 ± 0.15 ^b^	−3.85 ± 0.16 ^a^	88.76 ± 0.94 ^b^
SPI-E17.5	75.05 ± 0.51 ^d^	4.97 ± 0.48 ^c^	93.16 ± 0.37 ^d^
SPI-E22.5	72.84 ± 0.58 ^f^	7.52 ± 0.54 ^e^	93.94 ± 0.04 ^d^

Data are reported as mean ± standard deviation. Different letters indicated significantly different means (*p* < 0.05).
